# Keeping Lectures Alive in Undergraduate Medical Education: Current Status, Evolution, and Future Goals

**DOI:** 10.7759/cureus.87784

**Published:** 2025-07-12

**Authors:** Beatrice C Thomas, Georgina C Tiarks, Ghaith Al-Eyd, Vijay Rajput

**Affiliations:** 1 Department of Pediatrics, Cincinnati Children's Hospital Medical Center, Cincinnati, USA; 2 Department of Internal Medicine, The George Washington University School of Medicine and Health Sciences, Washington, D.C., USA; 3 Department of Medical Education, Nova Southeastern University Dr. Kiran C. Patel College of Allopathic Medicine, Davie, USA

**Keywords:** didactics, education, lecture, medical school, undergraduate medical education

## Abstract

Lectures have been the cornerstone of education for centuries. As society has modernized, science has elucidated multiple key aspects required for effective learning and retention of knowledge. In the last several decades, undergraduate medical education (UME) has changed with the incorporation of mandatory active small group learning and self-directed study in pre-clerkship education. Lectures have become non-mandatory school activities with poor attendance by students and a sore subject by the UME community. In addition, the use of standardized national exams throughout medical school has paved the way for the use of third-party commercial resources focused on “high-yield” materials that are widely supported by students and schools. Consequently, medical students have begun to forego traditional didactics in favor of commercial resources and self-study time. Hence, there is a growing frustration among faculty over poor attendance and engagement. However, lectures and seminars have continued to thrive in clerkships, graduate medical education, and continuing medical education despite the downfall in pre-clerkship education. Lectures in pre-clerkship education may need to be restructured and delivered in a style that fits the growing educational needs of advanced adult learners. This article critically reviews the historical role and current status of lecture in UME, while providing recommendations for future actionable changes.

## Introduction and background

Background

The overarching goal of undergraduate medical education (UME) is to graduate physicians competent enough to continue their training in the medical specialty of their choice. During UME, students study basic and clinical sciences to understand disease and apply this knowledge in clinical settings. Lectures have long been a cornerstone of teaching in both traditional discipline-based and integrated organ system-based curricula. However, the evolving need to emphasize active learning, including self-directed and lifelong learning, in UME has marginalized the role of traditional lectures by introducing a variety of active learning methods [1].

Lectures have been at the cornerstone of education for over 800 years [2]. Historically, lectures played a central role in the dissemination of scholarly knowledge. The term lecture originates from Latin, meaning to read or read aloud [3]. Before the printing press, books were scarce [3]. Students had no way of receiving their own copy of a book other than simply transcribing what was read aloud to them [2]. Consequently, this was the beginning of lecturing. The lecture methodology was used as a method to bridge written text and oral communication.

Lectures have survived for centuries due to their adaptability and versatility. One early evolution was the use of commentaries, summarized notes written in the margins [3]. Another great shift came in the 20th century alongside technological advancements. Overhead projectors were introduced after the Second World War [2]. Soon after, audio, video, teleprompters, and slide presentation software, such as PowerPoint, followed. In the modern-day 21st century, lectures encompass all aspects of education from kindergarten through graduate studies. Their flexibility and malleability have allowed lectures to transcend time and continue to be a presence in society as a learning method centuries after they were first used. Despite the durability of lecture-based teaching throughout history, there still exist multiple facets in which the traditional lecture can be improved to benefit the modern-day learner.

## Review

Learning principles

So why do we go to lectures, and school, in the first place? The answer is simultaneously simple and complicated; we go to learn and acquire knowledge that is not naturally gained. According to Sweller 2006, there are two distinct types of knowledge: primary and secondary [4]. Primary knowledge is gained simply by interacting with our environment, honed by centuries of evolutionary necessity. It consists of a wide spectrum of basic survival skills, e.g. verbal communication, social cues, and general problem solving. However, it is the acquisition of secondary knowledge, like reading and writing, that has led to schooling. This knowledge base has only become relevant over the past few millennia, with the development of science, mathematics, and literature. Evolution has not had enough time to develop strategies for easy and spontaneous learning of these concepts [4].

There are five basic principles of learning, each helping to ensure the durability of new information: the information store, the borrowing and reorganizing, the randomness as genesis, the narrow limits of change, and the environmental organizing and linkage [4]. The information store principle allows us to store information indefinitely. According to this principle, encoding new learning information depends on creating connections with prior experiences. The borrowing and reorganizing and the environmental organizing and linkage principle acknowledge that all newly learned information has first been borrowed or learned from another individual and allows for linkage with previously learned information, creating networks. The creation of these networks and newly learned information is then able to be transferred from short-term memory to long-term memory and recalled at a later date. These first four principles begin to explain the importance of proper teaching styles. Knowledge is dependent not only on prior experiences but also on the person teaching them. Teachers represent a formidable force in an individual’s learning process and can quite literally make or break how one learns a topic.

The final two principles help to explain why certain information is retained or lost, and how this process is different for every person. The randomness as genesis principle allows an individual to create and learn new information while problem solving. This principle is highly dependent on each person’s skill set. Not every student arrives at class with the same foundational problem-solving skills. The narrow limits of change principle places time constraints on learning multiple new concepts at the same time. If too much information is presented at one time, only some of it will get encoded into long-term memory. Together, these five principles underlie the basic tenets of short-term, long-term, and working memory and how an individual is able to access and use prior knowledge (Table 1). 

**Table 1:** Learning principles and examples in UME UME, undergraduate medical education

Principle	Definition	Example
Information store principle	Long-term memory of prior concepts	Recalling basic math skills when solving pharmacokinetics equations for step 1.
Borrowing and reorganizing principle	The process by which individuals attain new information from another source, combine it with prior knowledge, and store the information in long-term memory	A medical student is learning about cardiac physiology from their lecturer. They recall that their uncle was previously on sacubitril/valsartan for his heart failure. Subsequently, link these two concepts and are better able to recall the underlying mechanism of sacubitril/valsartan for treating heart failure.
Randomness as genesis principle	The creation of new information and novel concepts through problem solving	When taking an exam, a student is presented with a third-order question, which requires them to remember a disease process and then determine the outcome of a novel drug on the disease process.
Narrow limits of change principle	Inborn constraints of working memory capacity	A medical student only recalls select information from a recent lecture on inborn errors of metabolism.
Environmental organizing and linking principle	The process by which a person pulls stored information into working memory	A medical student is asked to present an interesting case during pediatric rounds. They are quickly able to parse through recent cases and present a case on Kawasaki Disease, remembering information from past lectures.

A few basic principles should lay the foundation of each learning opportunity. Information needs to be relayed clearly, students need to be paying attention, and the presentation should focus only on the relevant information [5]. Teaching for long-term retention requires the educator to employ multiple strategies to cater to individual needs. Vaughn and Baker report that educators should employ various approaches in their lecture styles to appeal to different needs [6]. The use of different visual and auditory aids and repetition of prior material can help a greater number of students learn the material.

According to the environmental organizing and linkage principle of learning, the educator should create connections between new knowledge and old knowledge. This could be done through case presentations to connect real-life scenarios to textbook material. Moreover, the randomness of genesis principle discusses the usefulness of problem-solving during the learning process. Therefore, including problem-solving tactics when teaching may help to solidify and retain material. Optimal learning occurs when students are steered to engage by applying newly learned concepts to solve relevant problems and store the related gained knowledge in long-term memory ready for similar applications in the future [4,6].

In addition, educators need to understand the basic limits of working memory. While our working memory can hold five to nine items at a time, we are unable to process more than four items simultaneously [7]. Therefore, presenting clear, succinct information within a shortened time frame may help to improve teaching and retention of material.

Memorable lectures

Everyone has experienced both engaging, riveting lectures and monotonous, subpar lectures. There is an array of reasons why a lecture may be touted as effective versus ineffective. However, one thing that can be agreed upon is that well-organized, stimulating lectures paired with a skilled lecturer are extremely beneficial to learners. Lecturing has grown throughout history to no longer be based on reading a book aloud but, instead, to also require a certain level of enthusiasm to engage the audience [8]. Lecturers have become performers and public orators [2]. This is what differentiates lectures from written text. Anyone can simply read a textbook, but the ability to creatively, originally, and engagingly explain a concept is an art [3].

Afzal and Barbar describe three key elements to memorable presentations: cognitive imprinting, simplification, and rehearsal of information [9]. Cognitive imprinting allows our brains to rapidly learn and memorize a piece of information. As information is imprinted, it becomes readily available for use and can more effectively be stored long-term. Information can be better imprinted through making deeper connections and stimulating interest in a topic. An example of this may be to include real-world cases to show the importance of the subject matter. Simplification is another key element to improve learning. This is what differentiates attending a lecture from reading a chapter from a textbook. Especially in medical education, lecturers have the hard task of explaining complex topics in a short period of time. If educators can break down the information and lower the cognitive load, learners are likely to retain that information. This may include utilizing entertaining mnemonics, teaching information in a stepwise fashion, and relating the topic to something already well understood. Finally, rehearsal of information is the third and final element of noteworthy lectures [9]. By asking questions, students will have to constantly retrieve that information, which makes the brain more likely to change structurally, chemically, or physically to integrate that information into long-term memory [9].

Ultimately, engaging the audience is another essential component of lectures. A study by Lenz et al. describes the benefit of electronic audience response technology to help engage students and increase recall by asking questions throughout [10]. Features like animations, videos, audio records, and images may also be used to capture the attention of the audience [10]. In addition to appropriate technology use, public speaking skills are one of the most effective methods of engagement. Familiarity with the lecture content, looking at the audience rather than the screen, and paraphrasing are all elements of public speaking that may be employed when lecturing. Even TED Talks are seen as contemporary, exciting seminars due to their conversationalist approach rather than reading verbatim off slides or notes. Promoting discussion in the audience through planned pauses and opportunities for peer-to-peer learning can also be used to captivate students [10].

In recent years, various modalities of active learning including those of self-directed and lifelong learning, such as problem-based learning (PBL) and just-in-time teaching (JiTT), have become popular in UME. The use of self-guided problem solving has been hailed as a novel approach to learning. Lectures, conversely, have been condemned during this same period for not promoting independent and critical thinking skills due to the passive learning environment [8]. The constructionist theory states that knowledge cannot simply be transferred person to person but, instead, must be actively worked on [8]. PBL forces students to actively seek information rather than passively absorb it during lectures, and JiTT allows instructors to identify learning gaps based on pre-class assignments [11]. Subsequently, they are pushed to utilize this knowledge to solve problems they may encounter in real life. Schmidt et al. also claim that an underlying issue with lectures is that they are dependent on the transmission fallacy [12]. Information cannot just be heard and placed into long-term memory; it must be recalled and applied to facilitate long-term storage. Another common problem with lectures is that few educators receive formal presentation or public speaking training [10]. Lecturers read off slides directly rather than paraphrasing, and in turn, do their students a disservice by regressing to the origins of lecture when they simply read verbatim. When educators have text-heavy presentations and fail to further elaborate on concepts other than what is written, they are failing to reduce cognitive load and provide deeper connections to the material.

In addition, constant technology use has increased students’ concentration threshold requiring a higher stimulus to keep their attention. One study found that students cannot focus for more than 15 minutes at a time [12]. Most lectures last for an hour with only a short break in between. This contradicts both the narrow limits of change principle and the cognitive load theory, which concurrently state that students can only process so much information at once [4].

Finally, the social networking and mentorship opportunities gained during in-person class time cannot be overstated. Mentorship is a valuable relationship between faculty and students, which can help to transform trainees into accomplished, experienced professionals [13]. This can be through career guidance, application tips, skills development, introductions to colleagues, or through volunteer and research opportunities. Mentors may also support medical trainees through personal aspects of medicine, such as difficult patient encounters or talking through poor outcomes [13]. Lectures allow students to find and develop relationships with faculty members, which can serve as a valuable source for mentorship.

In summary, to enhance student engagement and learning, lectures can be restructured into multiple objective-based short sections that introduce topics through relevant image- and illustration-based questions, supported by knowledge application exercises with embedded triggers that promote interactive problem-solving discussions.

Discussion

Although lectures have been a cornerstone of education for centuries, medical students are increasingly absent from their school’s lecture halls and instead turning toward commercial online resources. The Association for American Medical Colleges reported in 2019 that only 10% of students attend lectures “often” while 29% claim that they “almost never” attend [14]. The answer likely lies in the way the American medical students are being tested. While medical schools vary in their structure, each student’s education centers on two basic knowledge bases: the preclinical and clinical curricula. Traditionally, the preclinical curriculum utilizes lectures to help students acquire the fundamental knowledge of basic sciences principles, while the clinical curriculum occurs in a clinical setting. Following the preclinical and clinical years, students are required to take high-stakes national standardized exams (United States Medical Licensing Examinations known as USMLE) to determine whether they have retained knowledge that the medical community deems relevant. These standardized test outcomes are a critical component of their applications to competitive residency programs for their UME; an exam that students believe will make or break their career [14].

Lectures in the preclinical curriculum are increasingly competing with third-party digital resources that stress “high yield” topics. Put succinctly by Prober and Heath in their 2012 article regarding lectures in medical education, in the past 100 years “the volume of medical knowledge has exploded… [yet] the hours available in the day have not increased to accommodate the expanded medical canon” [15]. The evidence cannot be overstated; simply compare the length of first aid for the USMLE step 1 from 2001 to 2024. The book has nearly doubled, increasing from 481 to 848 pages [16]. Despite the vast increase in information, medical students are still expected to develop a baseline understanding of the current medical knowledge in just four years. To make matters more difficult, students are also expected to maintain adequate extracurriculars in order to match into residency, with the average number of publications tripling from 2007 to 2020 [17]. With this knowledge, it can be much easier to understand the mindset students have when it comes to attending lectures.

Lawrence et al.’s study of 58 second-year medical students highlighted this exact conundrum. When asked why they chose not to go to the lecture, one student highlighted, “Lectures have a lot of extraneous information that is neither clinically relevant nor even covered on the test. I know some people have intellectual curiosity about experiments and things like that. I personally don’t. I find it a really annoying experience to listen to a lecture where someone is going off on a ton of tangents or having classmates who are asking a bunch of tangential questions. It feels like a big waste of time when I could watch one of these videos in 10 minutes and get to the core facts. And that is a real benefit to how well organized these videos are” [18]. Unfortunately, with the constraints placed on students, they often have to cut corners and make decisions regarding where their time is best spent. And while students feel pressured to attend lectures to appease the feelings of the professors, many choose to utilize outside resources or recorded lectures as a means to save time. Students often cite lectures’ lack of “clarity, organization, efficiency, and style” as reasons why they choose other resources [18]. Current medical students have had technology ingrained into their everyday lives since birth [19]. So, when lectures fail to provide them with succinct, easily digestible, and engaging content, many students have begun to go in search of other options. Succinctly stated by one student, “I have to make choices between learning certain material and keeping up with my research or with an extracurricular” [18]. With only 24 hours in a day, it is no wonder that attendance rates in lectures have declined, with 28.8% of second-year students across the country admitting to “almost never” going to lecture [20].

To complicate matters even further, medical education varies drastically from a typical education system. The USMLE provides medical schools with outlines of the material to be tested, but it rests in the hands of each medical school to ensure that the proper breadth and depth of knowledge is provided to the students. It is also school-dependent as to how medical students will be assessed prior to taking these exams. Some schools elect to create in-house examinations, created by faculty, while other schools choose to create examinations using retired board questions provided by the National Board of Medical Examiners. Each evaluation strategy presents pros and cons. In-house examinations allow schools to tailor questions to the material that was directly presented to students during lectures. This is where commercial online resources offering standardized, shortened, and efficient information come into play. Not only can the students ensure that they are catering their knowledge base toward their upcoming licensing exam, but they are also saving time by using two-in-one resources for content and tests closely related to the board exam style and expectations.

However, the emergence of third-party resources has not occurred without controversy. In a career dominated by the expectations of professionalism, students often struggle to balance the expectations of their medical school faculty with the time constraints of a well-balanced life. For the student who attends the lecture, it is a common occurrence to wait for their professor, only to watch as the smile drops off the lecturer’s face as they stare into the abyss of chairs. It has to be acknowledged that many medical school professors have entered to the profession to enhance the lives of students and with the endurance of lectures within system, it stands to reason that many of these professors likely learned medicine solely through lectures and, therefore, may believe that lecturing is the best way of transmitting knowledge. So, when the professor walks in and sees the poor attendance rate mirrored at many medical schools throughout the country, it is understandable that some professors see the lack of attendance as “an egregious violation of professionalism” [21]. Moreover, professors believe that attendance at lectures provides the opportunity for students to develop professional relationships [22]. Medicine still revolves largely around networking and these relationships made during class time can be influential on a student’s later career. Medical educators want to interact with their students and pass on the knowledge that has helped them become excellent practitioners. However, traditional lectures might not be the best method for doing this. Increasingly, evidence-based medicine (EBM) has been integrated into medical school curricula, and its principles have been included in the design of many courses, including those primarily focused on patient care competency. Sackett et al. clearly defined the role of EBM in patient care as “the conscientious, explicit, and judicious use of current best evidence in making decision about the care of individual patients… it means integrating individual clinical expertise with the best available external clinical evidence from systematic research” [23]. How is it, then, that medicine has failed to adopt teaching strategies that have continuously been shown to yield better outcomes than the traditional lecture-based approach? Even when compared with other science, technology, engineering, and mathematics (STEM) fields, medicine has been “slow to adopt evidence-based practices for instruction that do not include lectures as a teaching learning strategy” [24]. A 2014 study looking at undergraduate STEM students highlighted that students in traditional, lecture-based learning settings were 1.5 times more likely to fail than their counterparts who participated in active learning methods [25]. Medical education also largely caters toward adult learners, who have the self-concept “of being responsible of their own decisions and own lives” [26]. Adult learners are goal-oriented and best learn through engagement in relevant learning experiences [27]. Knowing this, there exists a space within medicine to allow professors to adapt their lecturing approach to the emerging needs and learning styles of their students. For example, designing interactive case-based didactic discussions where faculty share their knowledge in an inspiring and invigorating way while establishing effective educational relationships with students. Alternatively, lecturers could offer the choice of written material (e.g. textbook or articles) or relevant videos to students as pre-work These interactive sessions may include application of knowledge problem solving activities, e.g., mini vignettes with embedded triggers that engage students in discussions to address applications of relevant basic and clinical science concepts into clinical reasoning. After all, medicine has always straddled the line of old and new: favoring technological advancements that improve patient outcomes while always staying true to the importance of a thorough physical exam, a practice that has existed in medicine since its inception. The ways to teach medical students should be no different.

However, the way in which the academic community embraces these active learning approaches could be its immediate downfall. Many medical professors who enter the classroom do so due to their love of educating the next generation of doctors, and seeing an enthusiastic response from their students provides “powerful, immediate feedback” as to whether they are establishing these profound connections [21]. Attendance is required for professors to establish these connections and feel the related gratification. It is likely safe to assume that dwindling attendance numbers in medical schools have led to blunted enthusiasm both among the professors and their lectures. Oddly enough, this same blunted enthusiasm can be found in students in regard to attending lectures. To many students, simply being “a passive receptacle waiting to be filled by factoids by faculty experts” does not inspire the fervor to attend lecture [28]. Some schools have tried to attack this conundrum by making lectures mandatory. Although this strategy will see classrooms filled, it likely fails to yield buy-in from students, leading to lower participation and lower long-term retention [29]. Alternatively, lecturers could include more active and engaging strategies in their lectures. Lectures that tell a story, keep slides simple, engage the audience through pauses and questions, and remain firmly within their allotted time are proven to increase learning [30]. Similar initiatives in the past have seen up to or greater than 95% attendance rates, despite remaining optional [24]. Students will utilize whatever resource they feel best prepares them for the exams that lie ahead of them. With careful thought, planning, and buy-in, lectures could soon see students returning to the classroom.

## Conclusions

Despite the emergence of commercial digital resources, there is still a fundamental role of lecture-based learning in the medical school curriculum. There is a reason lectures have persisted through millennia and still exist throughout every facet of academics. Death leads to a need-driven rebirth, and as such, there is space for lectures to reemerge in medical schools. Redesigning the lecture structure and delivery styles in line with the evolving needs of advancing UME and the principles of adult learning could motivate the students and save the lectures. Redesigns may include structuring session content into objective-based short sections that integrate relevant basic and clinical science concepts and apply related knowledge through interactive case-based discussions. However, for this rebirth to occur, there must be acceptance from both students and faculty to adapt lectures to the evolving landscape of UME.
